# Increased Latency of Visual Evoked Potentials in Healthy Women during Menstruation

**Published:** 2011-07

**Authors:** Mohsen Azarmina, Masoud Soheilian, Hossein Azarmina

**Affiliations:** Ophthalmic Research Center, Shahid Beheshti University of Medical Sciences, Tehran, Iran

**Keywords:** Visual Evoked Potential, Menstruation, Visual Cortex

## Abstract

**Purpose:**

To evaluate the latency of visual evoked potentials (VEPs) in healthy women during and after menstruation.

**Methods:**

Pattern and flash VEPs were performed in 15 healthy women aged 18 to 25 years on the maximum bleeding day (luteal phase) and 7 days after the menstrual cycle (follicular phase).

**Results:**

Mean latency was 119.6 msec on the maximum bleeding day and 100.8 msec one week after menstruation on pattern VEP (P < 0.001). Corresponding values for flash VEP were 124.5 msec and 112.7 msec, respectively (P < 0.001).

**Conclusion:**

Prolonged VEP latency on the maximum bleeding day indicates that high progesterone levels may have an inhibitory effect on optic nerve conduction velocity.

## INTRODUCTION

The visual evoked potential (VEP) is an electrical signal generated by the occipital visual cortex in response to stimulation of the retina either by light flashes or pattern stimuli. In cooperative subjects, the amplitude and implicit time of pattern VEP is less variable than flash VEP. On the other hand, the amplitude of flash VEP is greater and therefore more easily recorded. Flash VEP wave form, implicit time and amplitude are strikingly variable among different patients and even within the same subject. VEP can evaluate the integrity of the visual pathway and help in the diagnosis of optic nerve disorders.^1^,^2^ It has been reported that technical and physiological factors such as pupil diameter, refractive error, type of stimulus, age and sex, electrode position, and anatomical variations may affect VEP.^3^ In this study we evaluated VEP changes during and after menstruation, i.e., in luteal (high progesterone levels) and follicular phases (high estrogen levels), respectively.

## METHODS

The study included 15 healthy female volunteers with no systemic, gynecological or neurological disease. Informed consent was obtained from all participants. All subjects underwent a complete ophthalmologic examination; subjects with abnormalities in the retina or optic nerve, and those with refractive errors more than ±0.5 D were excluded.

VEP was performed in all subjects according to a standard method described by the International Society for Classification of Electrophysiology of Vision (ISCEV) using the MonElec2 system (Metrovision Inc., Pérenchies, France), on the maximum bleeding day and 7 days after cessation of menstruation, using both pattern reversal and flash light stimuli. For pattern VEP, stimulation was performed using 30 minute of arc alternating black and white check patterns on a video monitor at one meter with the subject fixating on a central target on the monitor. Flash stimuli were produced with a full-field (Ganzfeld) xenon arc photostimulator.

## RESULTS

Mean age of studied subjects was 20.7 ± 2.8 (range, 18 to 25) years. Latencies of pattern (P_100_) and flash (P_2_) VEPs were significantly higher on the maximum bleeding day in comparison with one week post-menstruation in all subjects (Table 1). Mean latency of pattern and flash stimulation decreased from 119.6 and 124.5 on the maximum bleeding day to 100.8 msec and 112.7 msec on the post-menstruation day on pattern and flash VEP, respectively (P < 0.001).

**Figure 1:**
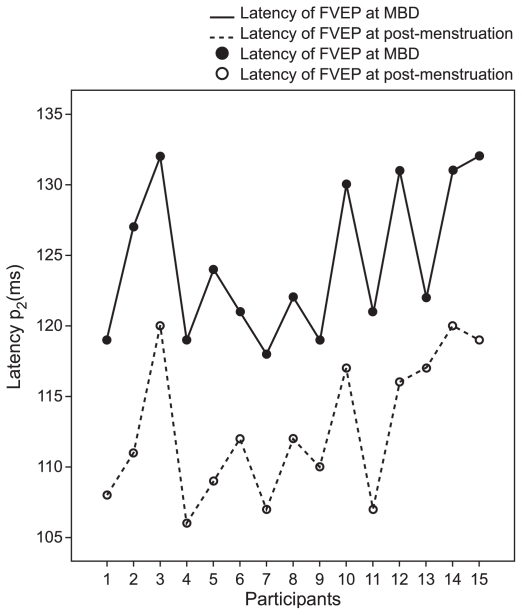
Distribution of latencies of flash visual evoked potentials on maximum bleeding day and on post-menstruation day.

**Figure 2:**
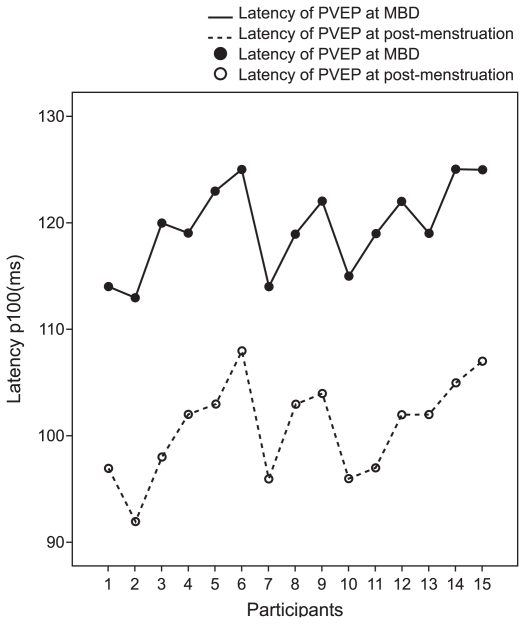
Distribution of latencies of pattern visual evoked potentials on maximum bleeding day and on post-menstruation day.

**Figure 3:**
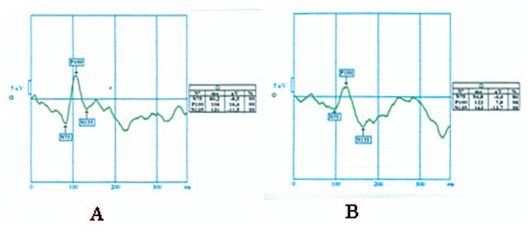
Latency of pattern visual evoked potential on post-menstruation day (A) and maximum bleeding day (B).

**Figure 4:**
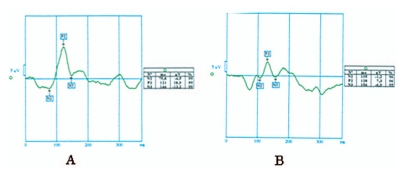
Latency of flash visual evoked potential on post-menstruation day (A) and maximum bleeding day (B).

The distribution of flash and pattern VEP latencies on the maximum bleeding day and on the post menstruation day are illustrated in figures 1 and 2 respectively. Figures 3 and 4 illustrate pattern and flash VEPs in a typical case on post-menstruation day and on maximum bleeding day, respectively.

## DISCUSSION

VEP is an evoked electrophysiological potential which can be extracted using signal averaging from electroencephalographic activity recorded at the scalp. Increased latency on VEP waves is the hallmark of many visual pathway diseases.^1^,^4^,^5^ There are different studies on VEP changes in healthy females during the menstrual cycle. Studying 23 healthy female subjects with regular menstruation, Kaneda et al^6^ showed increased latency on flash VEPs associated with low estrogen and high progesterone levels. Shushtarian et al^7^ also reported prolongation of flash VEP latency in 20 female subjects during a normal cycle. Furthermore, estrogen has been shown to shorten VEP latency in animals.^8^,^9^ Vingerling et al^10^ reported an association between macular degeneration and early menopause.^11^ The effect of estrogen on the central nervous system seems to be antagonized by progesterone and its metabolites, therefore prolonged VEP latency is thought to reflect the effect of progesterone.^8^–^10^,^12^

In our series, menstruation was associated with increased pattern and flash VEP latencies in 15 healthy women aged 18 to 25 years. The most probable reasons for increased VEP latency during menstruation may be as follows: (1) decrease in blood estrogen levels and diminution of the neuroprotective effect of estrogen^11^; (2) associated biochemical changes causing anxiety and stress^13^,^14^, thus interfering with concentration on the central target of the monitor; (3) vascular congestion around the optic nerve reducing conduction velocity.^12^,^15^

In conclusion, prolongation of VEP latency during the menstrual cycle in the luteal phase probably reflects the effect of progesterone. This effect is more notable on pattern as compared to flash VEP. The clinical implication of these findings is in the application of VEP for confirming demyelinative disease and optic neuritis. In such cases one should take into account that prolongation of VEP latency during menstruation may erroneously verify demylinating disease.

## Figures and Tables

**Table 1:** Latency of visual evoked potentials in 15 women on maximum bleeding days and post menstruation days

Subject	Age	Latency of PVEP (msec)		Lag of PVEP	Change (%)	Latency of FVEP (msec)		Lag of FVEP	Change (%)
	
		MBD	PMD			MBD	PMD		
1	19	114	97	17	17.5	119	108	11	10.2
2	18	113	92	21	22.8	127	111	16	14.4
3	18	120	98	22	22.4	132	120	12	10.0
4	22	119	102	17	16.7	119	106	13	12.3
5	25	123	103	20	19.4	124	109	15	13.8
6	18	125	108	17	15.7	121	112	9	8.0
7	25	114	96	18	18.8	118	107	11	10.3
8	18	119	103	16	15.5	122	112	10	8.9
9	20	122	104	18	17.3	119	110	9	8.2
10	20	115	96	19	19.8	130	117	13	11.1
11	18	119	97	22	22.7	121	107	14	13.1
12	19	122	102	20	19.6	131	116	15	12.9
13	24	119	102	17	16.7	122	117	5	4.3
14	22	125	105	20	19.0	131	120	11	9.2
15	25	125	107	18	16.8	132	119	13	10.9

Mean	20.7	119.6	100.8	18.8	18.7	124.5	112.7	11.8	10.5
SD	2.8	4.1	4.6	1.9	2.4	5.4	5.0	2.9	2.6
Median	20.0	119.0	102.0	18.0	18.8	122.0	112.0	12.0	10.3
Minimum	18.0	113.0	92.0	16.0	15.5	118.0	106.0	5.0	4.3
Maximum	25.0	125.0	108.0	22.0	22.8	132.0	120.0	16.0	14.4
95% CI for change		17.7 –19.9				10.2 – 13.4			
P		<0.001				<0.001			

PVEP, pattern visual evoked potential; MBD, maximum bleeding day; PMD, post-menstruation day; FVEP, flash visual evoked potential; SD, standard deviation; CI, confidence interval
